# Surgery-First Orthognathic Approach With 3D Virtual Planning: A Case Report

**DOI:** 10.7759/cureus.111015

**Published:** 2026-06-17

**Authors:** Meriem Bellamine, Amadou Oury Diallo, Meryem Lahlou, Tonamou François, Chaymae Chafik, Faiçal Slimani

**Affiliations:** 1 Dentofacial Orthopedics, Université Mohammed VI des Sciences et de la Santé, Casablanca, MAR; 2 Orthodontics and Dentofacial Orthopedics, Université Mohammed VI des Sciences et de la Santé, Casablanca, MAR; 3 Oral and Maxillofacial Surgery, Centre Hospitalier Universitaire Ibn Rochd, Casablanca, MAR

**Keywords:** 3d virtual planning, case report, dentofacial deformity, orthodontics, orthognathic surgery, surgery-first

## Abstract

The surgery-first orthognathic approach (SFOA) is an alternative to conventional orthognathic protocols, as it prioritizes skeletal correction before orthodontic treatment. This approach enables immediate aesthetic and functional improvement, making it an attractive option for the management of dentofacial deformities. This case report describes a 39-year-old patient with severe skeletal Class III malocclusion, anterior crossbite, mild mandibular crowding, and dentoalveolar compensation. Treatment was planned using three-dimensional (3D) virtual surgical planning to accurately determine maxillomandibular repositioning. Orthognathic surgery was performed without preoperative orthodontics and involved repositioning of both the maxilla and mandible. Early postoperative outcomes demonstrated significant aesthetic and functional improvements. Postoperative orthodontic treatment was initiated shortly after surgery to refine dental alignment and ensure proper arch coordination. The SFOA is a valuable option for carefully selected patients, offering reduced overall treatment time and enhanced patient satisfaction through immediate visible results. However, its success depends on accurate 3D planning and close collaboration between the surgeon and orthodontist.

## Introduction

Conventional orthognathic surgery traditionally requires prolonged preoperative orthodontic treatment to decompress the dental arches and prepare the occlusion for skeletal surgical correction. This preoperative phase may last 12 to 24 months, during which facial aesthetics may paradoxically worsen, negatively affecting patients' quality of life [1]. The surgery-first orthognathic approach (SFOA) represents a paradigm shift in the management of dentofacial deformities. This technique consists of performing skeletal surgical correction before any orthodontic treatment, followed by postoperative orthodontics to complete dental alignment and occlusion [2]. The modern concept of SFOA was systematized by Nagasaka et al. in 2009, who demonstrated the importance of a coordinated multidisciplinary approach between surgeons and orthodontists [3].

The potential advantages of the SFOA include immediate aesthetic and functional improvements, reduced overall treatment time, improved patient satisfaction, and accelerated postoperative orthodontic tooth movement due to the regional acceleratory phenomenon (RAP) [1,4]. The advent of three-dimensional (3D) virtual surgical planning has greatly facilitated the implementation of this approach by enabling precise prediction of occlusal and aesthetic outcomes [5]. However, careful patient selection is crucial for the success of the SFOA. Selection criteria include minimal anterior crowding, a favorable curve of Spee, a normally to moderately inclined incisor position, and minimal transverse discrepancy [4,6]. This case report illustrates the clinical application of the SFOA assisted by 3D planning.

Beyond facilitating the implementation of the SFOA, advances in 3D imaging and virtual surgical planning have transformed the diagnosis and management of dentofacial deformities. Conventional planning methods based on two-dimensional radiographs and model surgery are limited in their ability to accurately assess complex 3D skeletal discrepancies and facial asymmetries. In contrast, contemporary digital workflows integrating cone-beam CT, intraoral scanning, virtual surgical simulation, and computer-aided design and computer-aided manufacturing (CAD-CAM)-generated surgical splints allow for more precise visualization of craniofacial structures and more accurate transfer of the surgical plan to the operating room. Recent evidence suggests that virtual surgical planning provides improved soft tissue prediction and enhanced management of facial asymmetry compared with traditional planning approaches, contributing to greater predictability of surgical outcomes. Consequently, the integration of modern 3D technologies has become increasingly important in optimizing both functional and aesthetic results in orthognathic surgery [7].

Written informed consent was obtained from the patient for the surgical procedure and for the publication of this case report, including clinical photographs and radiological data, in accordance with current ethical guidelines and the recommendations of the institutional ethics committee. The patient’s identity has been protected, and all identifiable personal information has been removed.

## Case presentation

Clinical case

A 39-year-old patient, in good general health and with no relevant medical or surgical history, sought treatment for a dentofacial deformity. The patient specifically requested a rapid correction with immediate aesthetic improvement due to professional and social constraints. The consultation was motivated by several factors: aesthetic dissatisfaction with the facial profile and overall facial harmony; functional masticatory discomfort related to malocclusion; a negative psychosocial impact on self-confidence; and the desire for rapid aesthetic improvement without prolonging the preoperative orthodontic phase.

Diagnosis

**Figure 1:**
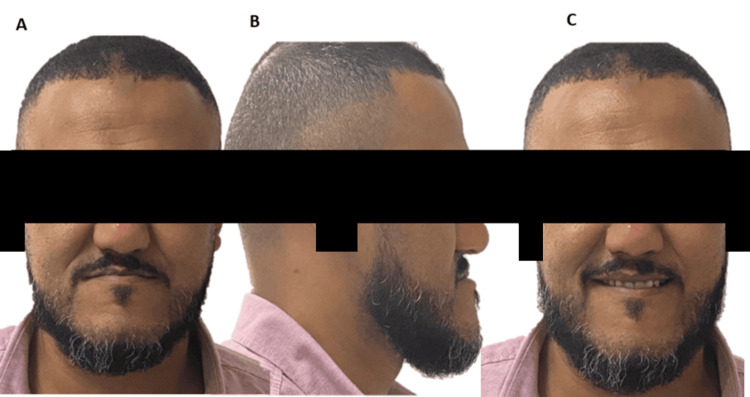
Preoperative facial photographs illustrating the facial aesthetic analysis (A) Frontal at rest. (B) Lateral at rest. (C) Frontal smiling

Facial analysis revealed a symmetrical long face with an increased lower facial third. A concave facial profile and a reduced nasolabial angle were observed (Figure 1).

**Figure 2:**
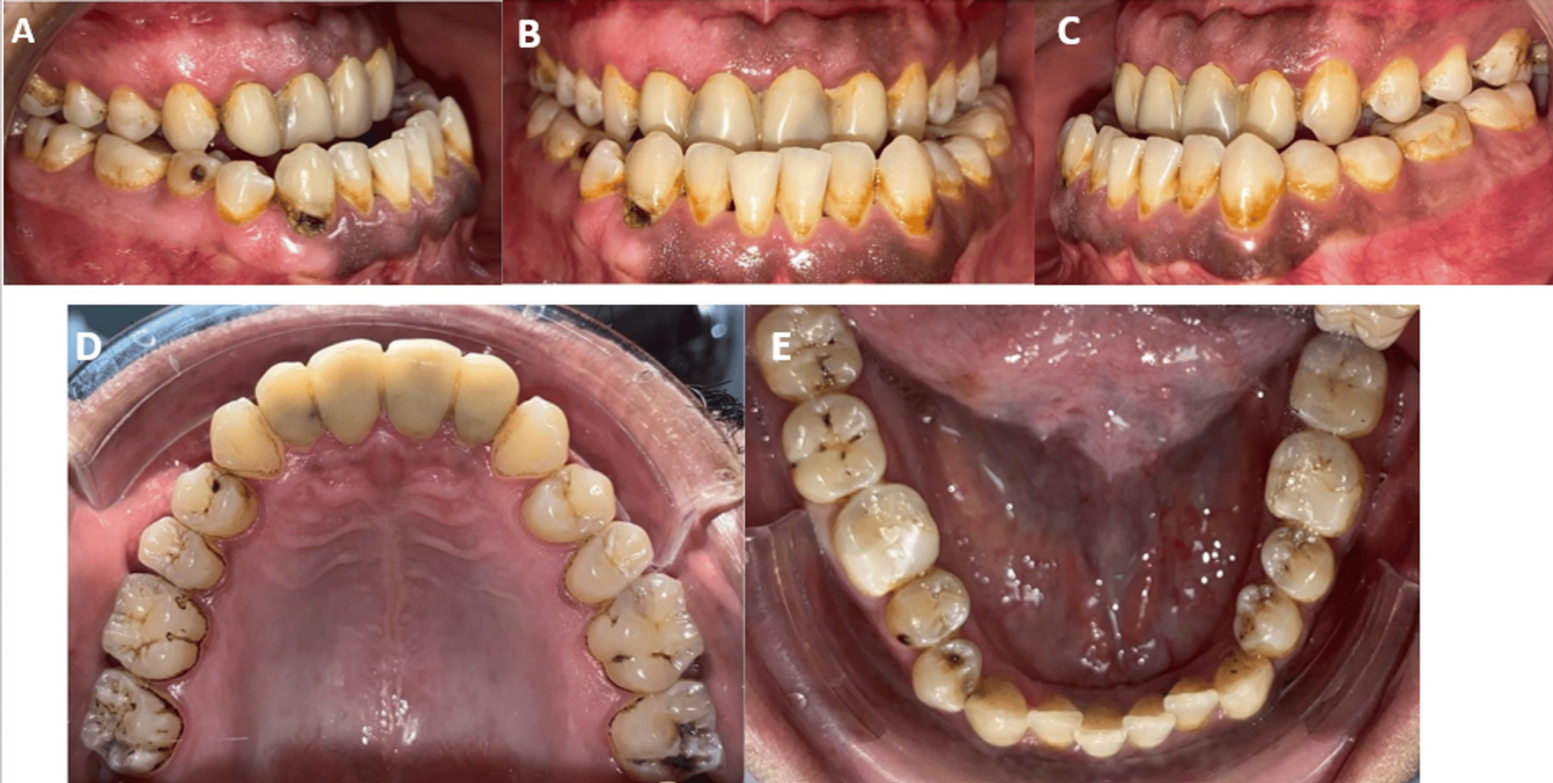
Preoperative intraoral photographs documenting the initial malocclusion (A) Right buccal view. (B) Frontal view. (C) Left buccal view. (D) Maxillary occlusal view. (E) Mandibular occlusal view

Intraoral examination revealed deviation of the interincisal midlines, anterior and lateral crossbite, and negative overbite. In the anteroposterior dimension, bilateral Class III molar and canine malocclusion was observed, with a negative overjet of -6 mm. Anterior mandibular crowding of 3 mm was present. The curve of Spee measured 2 mm. Retroclination of the mandibular incisors indicated dentoalveolar compensation. Oral hygiene was fair, with a thin periodontal biotype (Figure 2).

Preoperative paraclinical assessment

**Figure 3:**
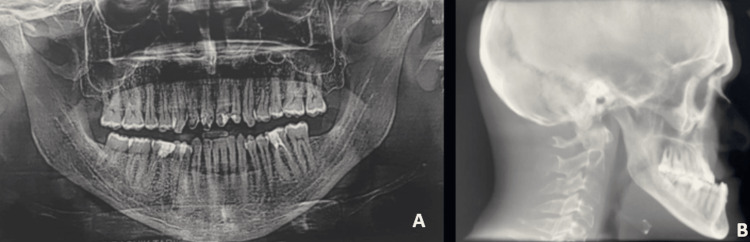
Preoperative panoramic radiograph and lateral cephalometric radiograph (A) Panoramic view. (B) Lateral cephalogram

As part of the preoperative assessment, a comprehensive radiological and digital evaluation was performed. Cone ceam CT (CBCT) provided a volumetric acquisition that enabled 3D analysis of the skeletal structures as well as evaluation of skeletal relationships in the three planes of space. In addition, the panoramic radiograph revealed an incomplete dental formula with the absence of the third molars (Figure 3). To further assess the dentofacial discrepancy, lateral cephalometric radiography was performed, and cephalometric analyses (Table 1) confirmed a skeletal Class III relationship with an ANB angle of -12° and an AoBo measurement of -16 mm. Finally, intraoral optical impressions were obtained through 3D digital acquisition of the dental arches, allowing virtual modeling and precise surgical planning.

**Table 1:** Pre-treatment cephalometric measurements FMA: Frankfort-mandibular plane angle; FMIA: Frankfort-mandibular incisor angle; IMPA: incisor-mandibular plane angle; SNA: Sella-Nasion-A point angle; SNB: Sella-Nasion-B point angle; ANB: A point-Nasion-B point angle; AoBo: A-point to B-point; I/NA: the angle and linear distance of the upper central incisor to the NA line, showing the inclination of your upper front teeth; I/NB: the angle and linear distance of the lower central incisor to the NB line, indicating how your lower front teeth are tilted; GOGN/SN: Gonion-Gnathion to Sella-Nasion angle

Measure	Normal	Pre-treatment
FMA	25° ± 3	40°
FMIA	67° ± 3	91°
IMPA	88° ± 3	65°
SNA	82°	83°
SNB	80°	95°
ANB	2° ± 2	-12°
AoBo	2 mm ± 2	-16 mm
I/NA	22°	33°
I/NA, mm	4 mm	7 mm
I/NB	25°	10°
I/NB, mm	4 mm	1 mm
GOGN/SN	32°	45°

Treatment objectives

The treatment objectives focused on both functional rehabilitation and facial aesthetic improvement. From an aesthetic perspective, the aim was to improve the nose-lip-chin relationship while correcting the sagittal and vertical skeletal discrepancies. Functionally, the orthodontic-surgical treatment sought to achieve a Class I canine relationship and establish a stable occlusion by obtaining functional anterior guidance with adequate overbite and overjet, thereby ensuring optimal masticatory function and long-term occlusal stability.

Treatment plan

An orthodontic-surgical treatment using SFOA was planned. Orthognathic repositioning splints were prepared through 3D virtual planning without preoperative orthodontic preparation.

3D virtual surgical planning

**Figure 4:**
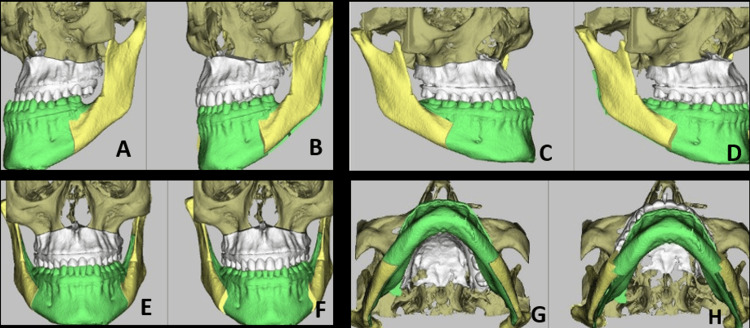
3D virtual planning showing preoperative analysis and predicted postoperative occlusion (A) Pre-planning left lateral view. (B) Post-planning left lateral view. (C) Pre-planning right lateral view. (D) Post-planning right lateral view. (E) Pre-planning frontal view. (F) Post-planning frontal view. (G) Pre-planning inferior view. (H) Post-planning inferior view

Three-dimensional virtual surgical planning was performed by merging CBCT data with intraoral digital models, enabling precise simulation of skeletal movements across all three planes of space. Based on this analysis, the surgical plan included a 4 mm maxillary advancement at point A, a 4 mm mandibular setback at point B, and a 3° clockwise rotation of the mandible. The predicted postoperative occlusion was subsequently evaluated virtually to anticipate potential occlusal interferences and optimize postoperative orthodontic management (Figure 4).

Treatment progress

Surgery was performed under general anesthesia with nasotracheal intubation. A bimaxillary osteotomy was carried out according to the SFOA protocol. The maxillary procedure consisted of a Le Fort I osteotomy performed through a maxillary vestibular approach with subperiosteal dissection. A horizontal osteotomy was made above the dental apices, followed by mobilization and repositioning of the maxilla in accordance with the virtual surgical planning. Stabilization was achieved using titanium osteosynthesis plates and screws.

**Figure 5:**
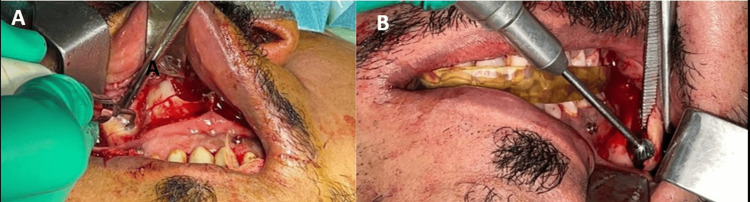
Intraoperative photographs showing Le Fort I osteotomy and BSSO (A) Le Fort I osteotomy cut. (B) Mandibular sagittal split osteotomy BSSO: bilateral sagittal split osteotomy

The mandibular procedure consisted of a bilateral sagittal split osteotomy (BSSO) of the mandibular ramus performed through a mandibular vestibular approach with subperiosteal dissection. The osteotomy line followed a modified Obwegeser-Dal Pont technique. Mandibular repositioning was guided by the surgical splint derived from the 3D planning, and fixation was achieved bilaterally using osteosynthesis plates and screws. Intermaxillary fixation was then performed using elastics attached to miniscrew anchorage. The total operative time was approximately three hours, and no intraoperative complications were observed (Figure 5).

The immediate postoperative management included multimodal analgesia consisting of paracetamol (1 g every six hours), non-steroidal anti-inflammatory drugs according to protocol, and opioids when necessary. Corticosteroid therapy was initiated with intravenous dexamethasone (8 mg) administered perioperatively, followed by a progressive oral taper over five days to control postoperative edema. Antibiotic prophylaxis was provided using amoxicillin-clavulanic acid (1 g/125 mg) three times daily for seven days.

**Figure 6:**
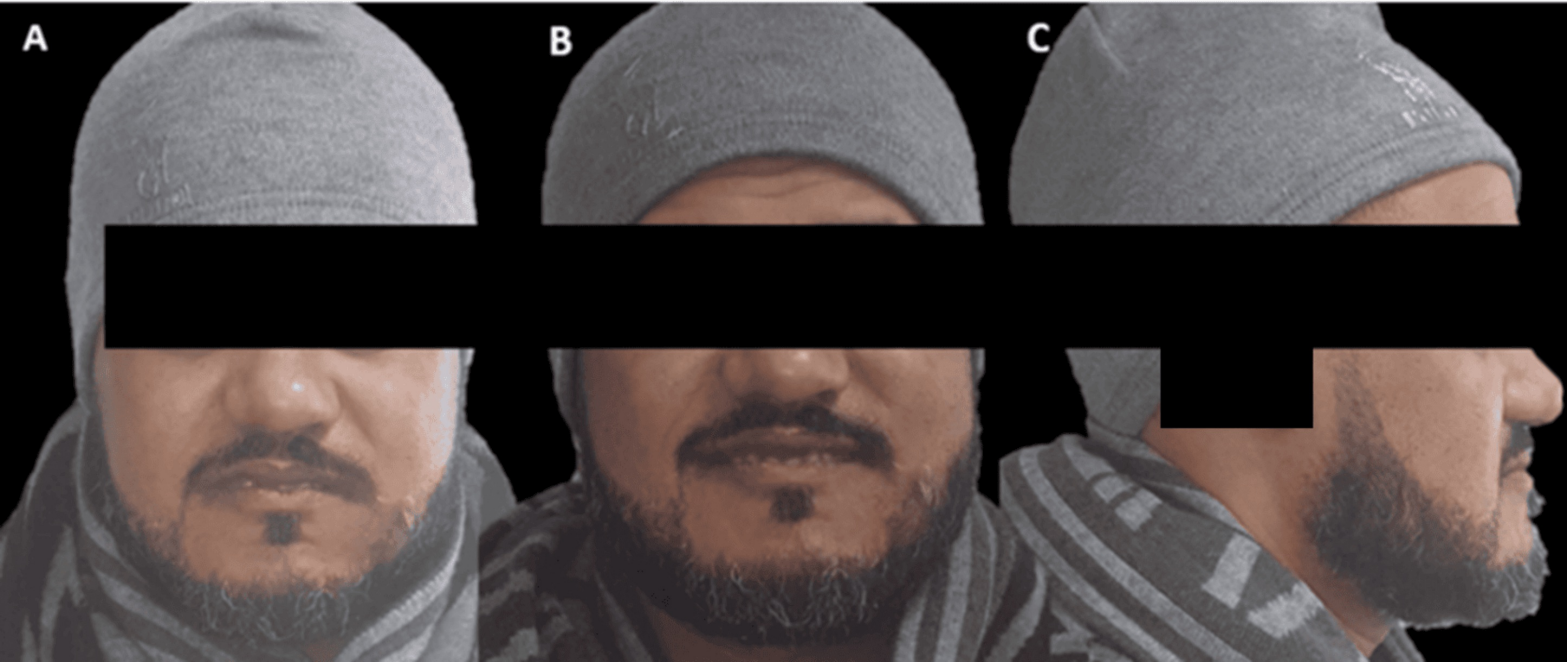
Facial photographs on postoperative day two showing immediate aesthetic improvement despite postoperative edema (A) Frontal view at rest. (B) Frontal smiling. (C) Lateral at rest

Postoperative oral hygiene measures included chlorhexidine 0.12% mouthwash three times daily, combined with careful tooth brushing. A blended diet was prescribed for four weeks, with gradual dietary progression thereafter. In addition, light intermaxillary elastics were worn at night for six weeks in order to guide occlusal healing and maintain postoperative stability. Clinical evaluation on postoperative day two demonstrated an improvement in facial aesthetics, with correction of the facial profile and satisfactory facial harmony. Postoperative pain was well controlled (visual analog scale (VAS) < 3/10), and postoperative edema remained moderate and was adequately controlled with corticosteroid therapy (Figure 6).

**Figure 7:**
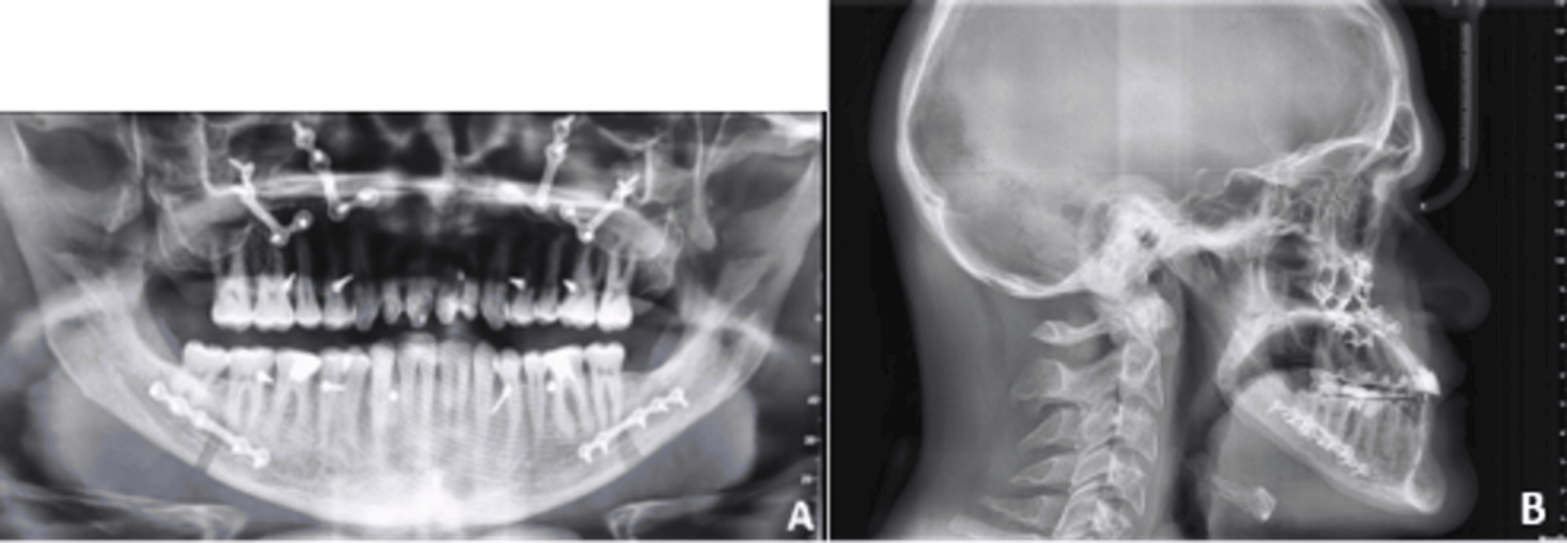
Postoperative radiographic records (A) Postoperative panoramic radiograph. (B) Post-surgical lateral cephalometric radiograph

Postoperative panoramic radiography suggested satisfactory positioning of the bony segments and osteosynthesis hardware (Figure 7A). Postsurgical cephalometric assessment indicated improvement in the skeletal relationship, with the ANB angle changing from -12° preoperatively to -3° postoperatively and the AoBo measurement improving from -16 mm to -5 mm. A reduction in vertical skeletal divergence was also observed, with the FMA decreasing from 40° to 35° and the GoGn/SN angle from 45° to 40° (Figure 7B, Table 2).

**Table 2:** Post-surgical cephalometric measurements FMA: Frankfort-mandibular plane angle; FMIA: Frankfort-mandibular incisor angle; IMPA: incisor-mandibular plane angle; SNA: Sella-Nasion-A point angle; SNB: Sella-Nasion-B point angle; ANB: A point-Nasion-B point angle; AoBo: A-point to B-point; I/NA: the angle and linear distance of the upper central incisor to the NA line, showing the inclination of your upper front teeth; I/NB: the angle and linear distance of the lower central incisor to the NB line, indicating how your lower front teeth are tilted; GOGN/SN: Gonion-Gnathion to Sella-Nasion angle

Measure	Normal	Post-surgical
FMA	25° ± 3	35°
FMIA	67° ± 3	93°
IMPA	88° ± 3	65°
SNA	82°	91°
SNB	80°	94°
ANB	2° ± 2	-3°
AoBo	2 mm ± 2	-5 mm
I/NA	22°	34°
I/NA, mm	4 mm	9 mm
I/NB	25°	10°
I/NB, mm	4 mm	1 mm
GOGN/SN	32°	40°

Postoperative orthodontic treatment

**Figure 8:**
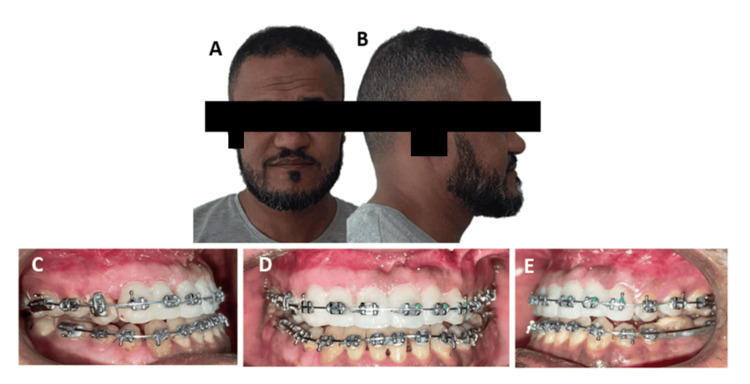
Extraoral and intraoral photographs at six-month follow-up showing the progression of postoperative orthodontic treatment and improvement of occlusion (A) Frontal at rest. (B) Lateral at rest. (C) Right buccal view. (D) Frontal view. (E) Left buccal view

Postoperative orthodontic treatment was initiated 30 days after surgery with the placement of a full fixed appliance on both arches using Roth prescription brackets with a 0.022-inch slot. The orthodontic phase aimed to achieve leveling and alignment of the dental arches, eliminate residual occlusal interferences, and ensure proper transverse coordination between the arches. Particular attention was also given to finalizing a functional occlusion with stable maximum intercuspation contacts as well as appropriate canine and incisal guidance. Long-term stabilization of the results was subsequently ensured through retention. Orthodontic activation began with light thermoactive nickel-titanium archwires (0.014 inch) for initial leveling and alignment, followed progressively by larger stainless steel archwires during the finishing phase (Figure 8).

Follow-up and outcome

**Figure 9:**
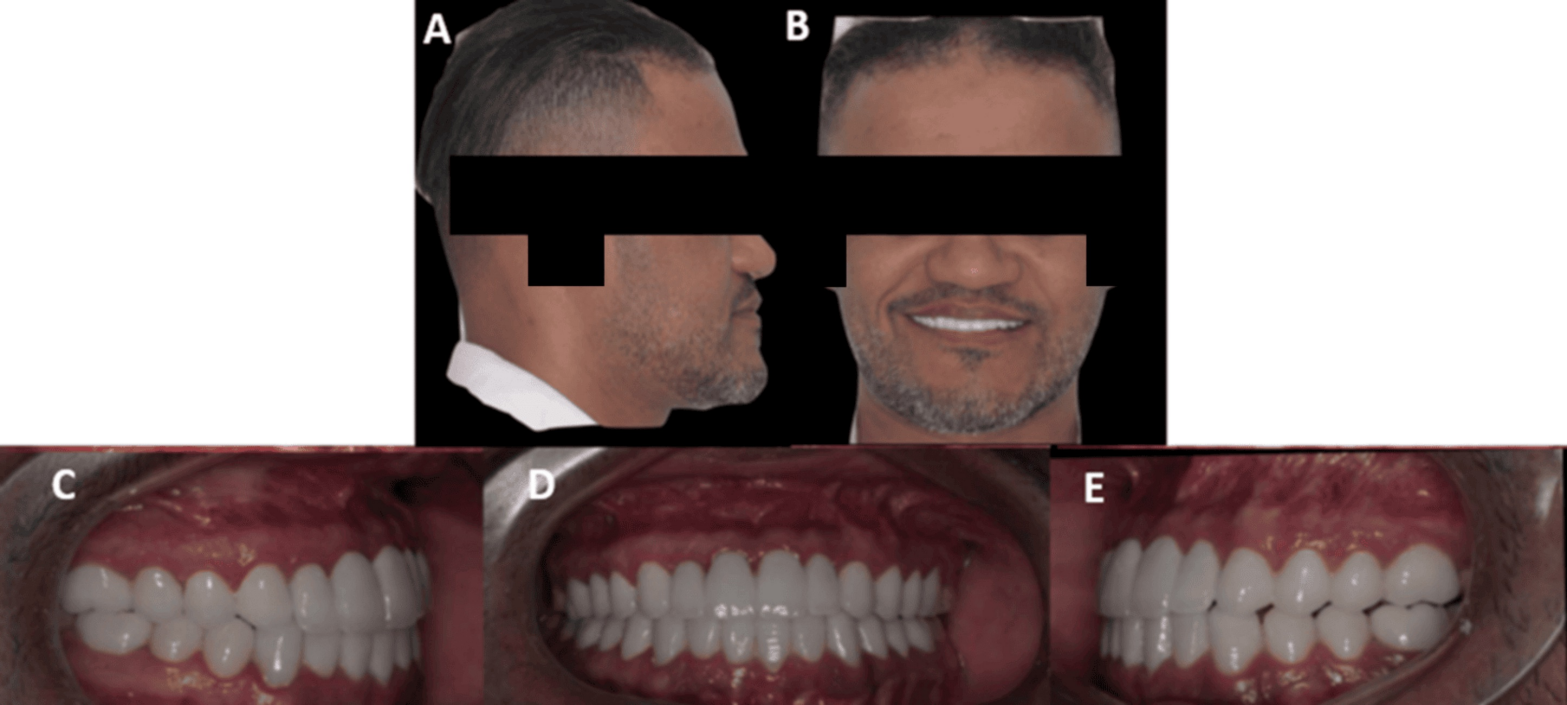
Extraoral and intraoral photographs showing the final results after prosthetic restoration (A) Lateral at rest. (B) Frontal smiling. (C) Right buccal view. (D) Frontal view. (E) Left buccal view

Postoperative orthodontic tooth movements progressed at an accelerated rate, which may be consistent with RAP [6]. Dental alignment progressively improved with a reduction in occlusal interferences. At the six-month follow-up, satisfactory arch coordination and occlusal settling had been achieved. The patient maintained good compliance with orthodontic treatment and postoperative recommendations. No clinical or radiographic signs of pathological root resorption were observed, and clinical assessment suggested satisfactory recovery of masticatory function. After debonding, the patient received ceramic veneers to correct dental discolorations and enhance smile aesthetics. Following prosthetic rehabilitation, retention was implemented using removable clear retainers worn at night to maintain the occlusal correction achieved and support the long-term stability of the treatment outcomes. At follow-up, the treatment outcomes appeared stable, and the patient reported satisfaction with the aesthetic and functional results (Figure 9).

The subjective perception of treatment success was further supported by objective patient-reported outcome measures. The Orthognathic Quality of Life Questionnaire (OQLQ) [8] was administered before treatment and at the six-month follow-up. The OQLQ score decreased from 85/88 before treatment to 3/88 at the six-month follow-up, reflecting a marked improvement in oral health-related quality of life. The most pronounced improvements were observed in facial aesthetics and social functioning, highlighting the positive psychosocial impact of immediate correction of the skeletal deformity (Table 3).

**Table 3:** Orthognathic Quality of Life Questionnaire (OQLQ) scores before and after treatment

Domain	Preoperative	Six-month follow-up
Facial aesthetics	20/20	0/20
Oral function	17/20	Jan-20
Awareness of facial deformity	16/16	Feb-16
Social aspects	32/32	0/32
Total OQLQ score	85/88	Mar-88

**Figure 10:**
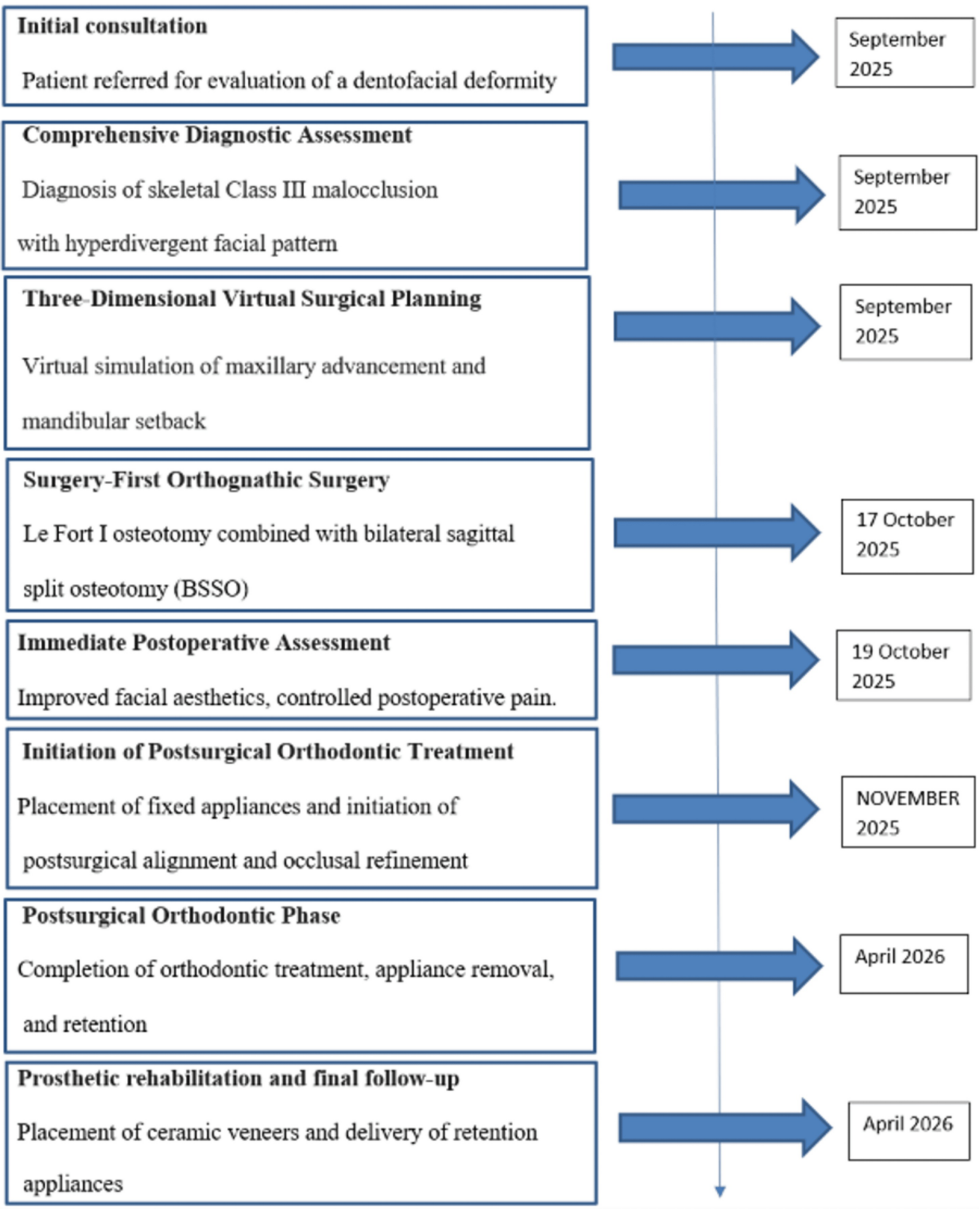
Timeline of the patient's treatment course

The sequence of treatment procedures, from diagnosis and three-dimensional virtual surgical planning to surgery-first orthognathic approach, postoperative orthodontic treatment, and final prosthetic rehabilitation, is summarized in Figure 10.

Treatment compliance and tolerance

Patient compliance throughout the various phases of treatment was satisfactory. Intermaxillary elastics were worn regularly according to the prescribed protocol. Monthly orthodontic appointments were attended, and oral hygiene was carefully maintained throughout follow-up. Tolerability of postoperative orthodontic treatment was generally good, with no major functional discomfort reported.

Adverse events

No major postoperative complications were observed. A transient bilateral hypoesthesia of the inferior alveolar nerve was noted in the immediate postoperative period, with complete progressive sensory recovery by month four. No signs of infection, suture dehiscence, pseudarthrosis, bone necrosis, or temporomandibular dysfunction were observed during follow-up.

## Discussion

This case report describes the clinical application of SFOA in the management of a skeletal Class III dentofacial deformity. The findings highlight several potential advantages of this approach as reported in the literature, while emphasizing the importance of appropriate patient selection and multidisciplinary treatment planning.

Discussion of relevant literature

The results of this case are consistent with the published data on the SFOA. The immediate aesthetic improvement and high patient satisfaction reported in this case correspond to the observations of Liou et al. [6], who documented significant psychosocial benefits of the SFOA compared with the conventional approach. The potential reduction in treatment duration, although not formally evaluated in this case, is supported by several studies reporting an average treatment duration of 12-18 months for the SFOA compared with 24-36 months for the conventional approach [1].

The accelerated postoperative orthodontic tooth movement observed clinically by the third month may be consistent with the RAP, which has been proposed as one of the biological mechanisms underlying the SFOA [6]. This phenomenon, characterized by a temporary increase in bone remodeling following surgical trauma, facilitates tooth movement during a four-to-six-month postoperative period. The 3D virtual planning used in this case reflects a major technological advancement in orthognathic surgery. Several studies have reported improved accuracy and predictability of this approach compared with conventional 2D planning in terms of the accuracy of skeletal movements and the predictability of aesthetic and occlusal outcomes [5]. In the specific context of the SFOA, this technology may play a particularly important role in compensating for the absence of preoperative orthodontic decompensation.

The favorable outcomes observed in this case may be related to several scientifically supported factors. The primary surgical correction of the skeletal discrepancy, which represents the underlying cause of the dentofacial deformity, may facilitate the immediate establishment of a more harmonious maxillomandibular relationship. This approach is biologically rational because it directly addresses the skeletal etiology rather than initially compensating with dental movements [4]. The cephalometric changes observed in this case further support the effectiveness of the surgical correction. The improvement in ANB and AoBo values reflected a substantial correction of the sagittal skeletal discrepancy, while the reduction in FMA and GoGn/SN angles suggested a favorable modification of the vertical skeletal pattern. These skeletal changes may have contributed to the establishment of a more balanced occlusion and improved facial harmony.

The patient's satisfaction may be related, at least in part, to the immediate aesthetic improvement, a concept supported by psychosocial studies in this field. The positive impact on quality of life from the early postoperative phase contrasts favorably with the conventional approach, in which a temporary deterioration of facial aesthetics is frequently observed during the preoperative orthodontic decompensation phase. Nevertheless, patient selection remains the determining factor for success. Patients presenting with severe dental crowding, significant dental compensation, or marked transverse discrepancy are not good candidates for the SFOA [4]. In such situations, preoperative orthodontic decompensation remains necessary to allow optimal skeletal repositioning and ensure postoperative occlusal stability.

Strengths of the case report

The main strengths of this case report lie in the rigorous patient selection, consistent with the criteria recognized in the literature for the SFOA, including minimal dental crowding, a favorable curve of Spee, and moderate dental compensation. The use of 3D virtual planning may have contributed to enabling precise prediction of outcomes and anticipation of postoperative occlusal challenges despite the absence of preoperative orthodontic preparation. Recent investigations have shown that virtual surgical planning combined with 3D-printed surgical guides allows clinically acceptable repositioning accuracy during bimaxillary orthognathic surgery, with mean linear deviations reported around 0.71 mm for the maxilla and 0.91 mm for the mandible, and mean angular discrepancies close to 0.95° [9]. These values highlight the high level of precision achievable with digitally guided orthognathic procedures.

Furthermore, the close multidisciplinary collaboration between the surgeon and the orthodontist from the planning phase played a key role in establishing a coherent therapeutic protocol and optimizing postoperative management. Finally, the immediate aesthetic and functional improvements may have contributed to the patient's reported satisfaction and adherence to postoperative orthodontic treatment.

Limitations

Although favorable outcomes were observed during the postoperative orthodontic phase, long-term follow-up after completion of treatment was not available. Consequently, the long-term stability of the skeletal, occlusal, and aesthetic outcomes could not be assessed. In addition, this report concerns a single, carefully selected case, which limits the generalizability of the findings to the broader population of patients undergoing orthognathic surgery. Finally, the absence of a comparison group treated with the conventional orthognathic approach precludes direct comparison regarding treatment duration, stability, and patient-reported outcomes.

Patient perspective

"When I first consulted, I was very concerned about my facial appearance and the impact it had on my professional and personal life. The medical team presented me with two options: the conventional approach requiring about two years of treatment, with an initial phase during which my appearance would temporarily worsen, or the surgery-first approach, which promised immediate improvement. Based on the information provided, I felt that the surgery-first approach was the most appropriate option for my situation. The possibility of obtaining immediate aesthetic improvement without going through a phase of deterioration was extremely appealing. The team took the time to explain the process, the benefits, and the potential risks in detail. The 3D virtual simulation really helped me visualize the expected results, which strengthened my confidence in the treatment. From the first days after surgery, despite the swelling, I could already see the improvement in my profile. This immediate improvement was an extremely important motivational factor in following the postoperative orthodontic treatment rigorously. I am satisfied with the results, both aesthetically and functionally. My only concern now is maintaining these results in the long term, and I am fully committed to following all the recommendations of the healthcare team."

## Conclusions

SFOA can be an effective treatment option for managing dentofacial deformities, providing immediate aesthetic and functional improvement while reducing overall treatment time, provided that it is supported by careful patient selection, precise 3D planning, and multidisciplinary coordination. It also reflects a shift back toward the original principles of orthognathic surgery, with greater emphasis on patient quality of life and rapid results. However, SFOA requires significant technical expertise, has a steep learning curve, and is not suitable for all cases. Limitations include challenges in predicting soft-tissue changes, as well as cost and accessibility constraints. Further consensus and clinical guidelines are needed to better define indications and optimize case selection.
